# Supplementary material for: Recapitulating human cardio-pulmonary co-development using simultaneous multilineage differentiation of pluripotent stem cells
Source: eLife. 2022 Jan 12;11:e67872. doi: 10.7554/eLife.67872 (PMC8846595; doi:10.7554/eLife.67872)
Supplement: Supplementary file 1. [file elife-67872-supp1.docx]

**Supplementary Information for**

***Recapitulating Human Cardio-pulmonary Co-development Using Simultaneous Multilineage Differentiation of Human Induced Pluripotent Stem Cells***

Wai Hoe Ng^1^, Elizabeth K. Johnston^1^, Jun Jie Tan^2^, Jacqueline M. Bliley^1,3^, Adam W. Feinberg^1,3^, Donna B. Stolz^4^, Ming Sun^4^, Piyumi Wijesakara^1^, Finn Hawkins^5^, Darrell N. Kotton^5^, Xi Ren^†,1^

**Authors’ affiliation:**

^1^ Department of Biomedical Engineering, Carnegie Mellon University, Pittsburgh, Pennsylvania, USA

^2^ Advanced Medical and Dental Institute, Universiti Sains Malaysia, Penang, Malaysia

^3^ Department of Materials Science and Engineering, Carnegie Mellon University, Pittsburgh, Pennsylvania, USA

^4^ Center for Biologic Imaging, University of Pittsburgh, Pittsburgh, Pennsylvania, USA

^5^ Center for Regenerative Medicine of Boston University and Boston Medical Center, Boston, MA 02118, USA

^†^ Correspondence

**Authors of correspondence:**

Xi Ren, PhD
Carnegie Mellon University, Scott Hall 4N111

5000 Forbes Avenue, Pittsburgh, PA 15213
Telephone: 1-412-268-7485

Email: [xiren@cmu.edu](mailto:xiren@cmu.edu)

**Supplementary File 1: Media Recipes/Composition**

| **Media** | **Base** | **Cytokines/Growth Factors** | **Final Concentration** |
| --- | --- | --- | --- |
| **Stage 1: Day 0 - 1** | mTESR Plus | CHIR99021 | 7 μM |
|  |  | Y27632 | 10 μM |
| **Stage 1: Day 2 – 3** | RPMI 1640 | Y27632 | 10 μM |
|  | B-27 minus insulin |  |  |
|  | GlutaMAX (1x) |  |  |
| **Stage 2: Day 4 – 7** | RPMI 1640 | A8301 | 1 μM |
|  | B-27 complete | IWP4 | 5 μM |
|  | GlutaMAX (1x) | Y27632 | 10 μM |
| **Stage 3: Day 8 – 14** | RPMI 1640 | CHIR99021 | 3 μM |
|  | B27 complete | Retinoic acid | 100 nM |
|  | GlutaMAX (1x) |  |  |
| **Stage 4: Day 15 -17** | RPMI 1640 | CHIR99021 | 3 μM |
|  | B27 complete | KGF | 10 ng/mL |
|  | GlutaMAX (1x) | Dexamethasone | 50 nM |
|  |  | cAMP | 0.1 mM |
|  |  | IBMX | 0.1 mM |
| **Stage 4: Day 18 onwards** | RPMI 1640 | KGF | 10 ng/mL |
|  | B27 complete | Dexamethasone | 50 nM |
|  | GlutaMAX (1x) | cAMP | 0.1 mM |
|  |  | IBMX | 0.1 mM |
